# Barriers associated with care-seeking for institutional delivery among rural women in three provinces in Afghanistan

**DOI:** 10.1186/s12884-018-1890-2

**Published:** 2018-06-18

**Authors:** Ariel Higgins-Steele, Jane Burke, Abo Ismael Foshanji, Farhad Farewar, Malalai Naziri, Sediq Seddiqi, Karen M. Edmond

**Affiliations:** 1 UNICEF Afghanistan, Kabul, Afghanistan; 2 ATR Consulting Afghanistan, Kabul, Afghanistan; 3 Afghanistan Ministry of Public Health, Kabul, Afghanistan; 4 United Nations Office Complex in Afghanistan (UNOCA), Jalalabad Road, Kabul, Afghanistan

**Keywords:** Maternal health, Institutional delivery, Skilled birth attendance, Barriers, Afghanistan

## Abstract

**Background:**

In the past fifteen years, Afghanistan has made substantial progress in extending primary health care. However, coverage of essential health interventions proven to improve maternal and neonatal health outcomes, particularly skilled birth attendance, remains unacceptably low. This is especially true for those in the poorest quintile of the population. This cross-sectional quantitative and qualitative study assessed barriers associated with care-seeking for institutional delivery among rural Afghan women in three provinces.

**Methods:**

The study was conducted from November to December 2016 in 12 districts across three provinces – Badghis, Bamyan, and Kandahar – which are predominately rural. Districts were used as the primary sampling unit with district-level sample sizes reflecting the ratio of that district’s population to provincial population. Villages within these districts, the secondary sampling units, were randomly selected. A household survey was used to collect data on: demographics, socio-economic status, childbearing history, health transport and service costs, maternal health seeking behavior and barriers to service uptake. Data on barriers to facility delivery were compared across provinces using chi square tests.

**Results:**

Of the 2479 women of child bearing age interviewed, one-third were from each province (33% *n* = 813 Badghis, 34% *n* = 840 Bamyan, 33% *n* = 824 Kandahar). Among those respondents who had delivered none of their children in a health center, money to pay for services appeared to be most important barrier to accessing institutional delivery (56%, *n* = 558). No transportation available was the second most widely cited reason (37%, *n* = 368), followed by family restrictions (*n* = 30%, *n* = 302). Respondents in Badghis reported the highest levels of barriers compared to the other two provinces. Respondents in Badghis were more likely to report familial or cultural constraints as the most important barrier to institutional delivery (43%) compared to Bamyan (2%) and Kandahar (12%) (*p* < 0.001).

**Conclusions:**

Despite the socio-demographic and geographic diversity of the three provinces under study, the top barriers to institutional delivery reported in all three areas are consistent with available evidence, namely, that distance, transport cost and transport availability are the main factors limiting institutional delivery. Proven and promising approaches to overcome these barriers to institutional delivery in Afghanistan should be explored and studied.

**Electronic supplementary material:**

The online version of this article (10.1186/s12884-018-1890-2) contains supplementary material, which is available to authorized users.

## Background

Afghanistan has among the highest maternal (396 per 100,000 live births) and newborn mortality (40 per 1000 live births) rates in the world [1, 2]. However, in the past fifteen years, Afghanistan has made substantial progress in extending primary health care through its ‘basic package of health services’ (BPHS) [3, 4] including improved availability of public facilities across the country [5]. Health facility deliveries (also called institutional deliveries) and birth assistance from a skilled attendant (midwife, nurse or doctor who has received training in midwifery or obstetrics) have increased dramatically from less than 15% for both indicators in 2002 [6], rising to 32% of births at a health center and 34% with a skilled attendant in 2010 [7]. By 2015, these indicators had further improved with 48% of births occurring at a health facility and 51% of births assisted by a skilled provider [8]. These figures and other figures indicate that almost all skilled birth attendants are assisting women at health facilities: 99.5% of deliveries in health facilities are assisted by skilled providers compared to 13% of births that occur elsewhere and 11% of births where location was not reported [8].

Coverage of essential health interventions proven to improve maternal and neonatal health outcomes, particularly skilled birth attendance, remains unacceptably low among populations in the poorest quintile (22%) as compared to the richest (82%) and the rate of change in overall improvements for this indicator appears to be decreasing [8]. In rural areas, only 40% of birth occur at health facilities, compared to 76% in urban areas, and only [8]. Global evidence suggests that the “best intrapartum-care strategy is likely to be one in which women routinely choose to deliver in a health centre, with midwives as the main providers, but with other attendants working with them in a team” [9].

Improvements have occurred unequally with considerable barriers to much of the population maintained [10]. Skilled birth attendance in Afghanistan is strongly associated with wealth of households, literacy, and residing less than one hour from a health facility [11]. Studies indicate that distance from health facilities, lack of transport, and high cost of transportation to go to a health facility are commonly cited as barriers to accessing primary health services in Afghanistan [12, 13]. Although all public health services are supposed to be free of charge across the country, households bear costs even in the public sector including for transportation, food, and medicines. According to Afghanistan’s National Health Accounts 2014, 73% of total health expenditure is comprised of household out-of-pocket expenses, and 7% of total household out-of-pocket expenditures is consumed by transportation related costs associated with health care [14].

The ‘three delays model’ represents the three main time-related factors that affect the outcome of an emergency presentation during pregnancy or child birth and are defined as: (i) delay in the decision to access care, (ii) delay in the identification of – and transport to – a medical facility, and (iii) delay in the receipt of adequate and appropriate treatment [15]. Women in Afghanistan experience all barriers, with the second delay being a particular challenge in rural areas. Additionally, Afghanistan is frequently classified as a ‘fragile’ state with high levels of ongoing conflict [16] and many women also lack power in the household to make decisions about health care for themselves and their children [17].

There are few studies which provide evidence on barriers to care-seeking for institutional delivery. The limited scope of available evidence combined with high concern for the low rates of skilled birth attendance and institutional delivery in the country merit additional investigation [18–20]. Some interventions, such as pay per performance of health facilities have shown minimal effect in Afghanistan, with inadequate attention to demand side factors as a possible reason. This study thus implemented a cross-sectional quantitative and qualitative study to assess the barriers associated with care-seeking among rural women who live in three disparate provinces in Afghanistan.

## Methods

### Design and setting

This study used a mixed methods approach – quantitative household questionnaire and qualitative data – to assess care-seeking behaviors and barriers to institutional delivery. The primary variables of interest were place of birth (facility or home) and reported barriers for facility-based birth. The household survey was organized in several sections or modules: respondent characteristics including socio-economic factors and childbearing history, maternal health seeking behavior, and service utilization. Given the nature of this study, including sensitivities around maternal in the country context, qualitative data is intended to complement and assist in explaining and interpreting quantitative findings.

The study was conducted from November to December 2016 in 12 districts across three provinces – Badghis, Bamyan, and Kandahar. Bamyan is a remote and rural mountainous province in the central Highlands region, Badghis and Kandahar are predominately rural with strong influence of anti-government groups, frequent conflict, and strict social norms. Badghis borders with Turkmenistan and has difficult and mountainous terrain, Bamyan is also very mountainous with remote settlements, while Kandahar is a southern region bordering Pakistan and is predominately flat and arid. The crude birth rate was 37 per 1000 population in rural areas; total fertility rate expressed per woman 16–49 years of age was 5.4 in rural areas compared to 4.8 in urban. The institutional delivery rates were 5.9% Badghis, 35.5% Kandahar and 46.2% Bamyan [8].

### Sampling and inclusion criteria

The sample size, per province, was determined to account for later disaggregation between control and treatment districts, for a planned endline study. The sample size was based on a simple random sample and did not take into account clustering, for a total of target size of 2400 households from twelve districts. The primary sampling units selected were districts pre-defined on the basis of comparability between assigned treatment and control districts within the three provinces. The population assigned per district was based on population proportional to size sampling. Villages, the secondary sampling unit were selected randomly from lists identified through a combination of participatory mapping and data provided by Afghanistan’s Central Statistics Organization. Eligible women respondents were selected randomly with approximately 20 households interviewed in each secondary sampling unit.

Respondents were selected through systematic random sampling using a ‘random walk’ protocol. A ‘random walk’ protocol was used instead of a household listing as there were no census data available [21]. Within urban areas, data collectors started at a central location, such as a mosque or school, stopping at every third house. Data collectors turned right at every corner to enable surveying of households off of the main roads. In rural areas, data collectors stopped at every third house. Within each household, eligible women who were married, aged 16–49 years and had had a still or live birth in the past two years were randomly selected from a household roster for quantitative interview. Women were purposively sampled for inclusion in the qualitative study based on eligibility per study group.

### Data collection

For the quantitative study eight female data collectors were recruited from each of the three provinces for training on quantitative and qualitative data collection methods. A household questionnaire was used to collect data from the interviewees on key characteristics: demographics, socio-economic status, childbearing history, health transport and service costs, maternal health seeking behavior, service use, and reported barriers (for data collection instrument, see Additional file 1). For logistics reasons, namely that female data collectors were at specific rural sites and trained in both methods, quantitative and qualitative data were collected simultaneously.

Surveys were conducted using Electronic Tablet devices enabled with KoBo Toolbox questionnaire software and data were collected using the tablet and automatically input skip patterns.

For the qualitative component, two methods were used – focus group discussions (FGDs) and key informant interviews (KIIs) – using semi-structured data collection guides. These guides contained domains relevant to the study, namely maternal care-seeking around birth and associated barriers. For FGDs, the respondent categories included females of reproductive age and community leaders; for the KIIs, respondent categories included service providers, specifically community health workers, midwives, nurses, and doctors. Each FGD consisted of between 6 and 8 women and lasted two to three hours. Major themes included public health service use in general, specific health behaviours related to maternal and newborn health, the role of community health workers, and barriers to both general and maternal and newborn health service uptake. The location of each FGD was determined through consultation with community leaders to provide a safe space for female respondents. In some areas, the location was a local school or mosque. In others, it was an individual’s house. The determination was based on local conditions including social norms, available space, and security. Recordings were transcribed and translated into English.

### Data analysis

Quantitative data were reviewed, cleaned, and analyzed in STATA 13. As the primary purpose of this study was descriptive, no groupings or stratification occurred besides provincial level analyses. Cross tabulations were calculated to identify possible relationships between key variables and the likelihood of at least one child being born in a health centre.

Badghis province is well recognised to be one of the most disadvantaged and conservative provinces in Afghanistan [8]. Thus we also wished to assess if there were important differences between the proportion of women reporting barriers to institutional delivery in Badghis province compared to Kandahar and Bamyan provinces. We used univariable logistic regression in Stata version 15 to conduct these analyses.

Qualitative data from 9 FGDs and 16 KIIs (Table 1) was coded by theme and sub-theme and analyzed in Atlas.ti in combination with a manual review. Before coding the data, an a priori code list was developed and during coding emergent themes were added. The major themes were identified based on the overarching research questions of the analysis and harmonized between quantitative and qualitative data collection tools. Qualitative data were required to add nuance and contextualize quantitative data. As qualitative data were not collected in all areas of study, there was no effort to compare district-level observations across qualitative and quantitative data. Rather, the qualitative data were used to triangulate findings from quantitative data and integrated into the analysis through summary of overall trends and observations.

**Table 1:** Sample by province and district for quantitative (including refusals and replacements) and qualitative (by type)

Province	District	Quantitative		Qualitative				
		Number of questionnaires administered (n)	Number of refusals/ replacements n (%)	Focus groups discussions		Key informant interviews		
				Females reproductive age	Community leaders	Community health workers	Doctors, Nurses	Midwives
Badghis	Qades	217	0	–	–	–	–	–
	Jowand	196	0 (0%)	1	–	–	1	1
	Abkamary	302	4 (1%)	–	1	1	1	–
	Moqur	100	0 (0%)	1	–	1	–	1
Bamyan	Punjab	312	0 (0%)	–	–	–	–	–
	Saighan	107	52 (49%)	1	–	1	–	1
	Waras	312	0 (0%)	–	1	1	–	–
	Kahmarad	109	2 (2%)	1	–	–	1	–
Kandahar	Arghandaab	159	5 (3%)	–	–	–	–	–
	Spin Boldak	258	28 (11%)	–	1	–	–	1
	Dand	228	1 (0%)	1	–	1	–	1
	Daman	179	5 (3%)	1	–	1	–	1
Total		2479	99 (4%)	6	3	6	3	6

### Ethical approval

Informed verbal consent was obtained from all participants in the study, and marked at the beginning of each questionnaire. The study, final research protocol and data collection tools received full ethical approval from the Ministry of Public Health Institutional Review Board (IRB) in Afghanistan (#335540).

## Results

### Socio demographics of the study population

A total 2479 women who had given birth in the last two years were approached for interview across the three provinces. There were 99 refusals. Women who refused to participate were replaced using the random walk protocol to select the next available house. Of the 2479 women interviewed, approximately one-third were from each location: 840 (33.9%) were from Bamyan, 815 (32.9%) were from Badghis and 824 (33.2%) were from Kandahar (Table 1).

The majority of women interviewed were between 20 and 30 years old, and most had limited access to formal education. Literacy rates among respondents were low in all provinces, with around 20% of the respondents indicating they were literate and less than 20% reporting having attended formal schooling (Table 2). Across the study population in the three provinces, while access to formal education appeared to be low, literacy rates did not directly correspond to formal education rates.

**Table 2:** Literacy rates, number of health facility visits, time and method to nearest health facility

	Overall	Badghis	Bamyan	Kandahar
	100% (*n* = 2479)	33% (*n* = 815)	34% (*n* = 840)	33% (*n* = 824)
Mother’s reported literacy				
Can read and write	20% (*n* = 508)	17% (*n* = 142)	23% (*n* = 192)	21% (*n* = 174)
Cannot read and write	79% (*n* = 1952)	82% (*n* = 670)	77% (*n* = 648)	80% (*n* = 634)
Don’t know	0% (*n* = 6)	0% (*n* = 3)	0% (*n* = 0)	0% (*n* = 3)
Refused	1% (*n* = 13)	0% (n = 0)	0% (*n* = 0)	0% (*n* = 0)
Number of health facility visits (last 12 months)				
No visits	6% (*n* = 158)	10% (n = 81)	5% (*n* = 41)	4% (n = 36)
1–5 visits	46% (*n* = 1144)	68% (*n* = 551)	32% (*n* = 268)	40% (*n* = 325)
6–9 visits	29% (*n* = 719)	7% (*n* = 61)	50% (*n* = 421)	29% (*n* = 237)
10+ visits	12% (*n* = 290)	1% (n = 6)	13% (*n* = 105)	22% (*n* = 179)
Do not know/ no response	7% (*n* = 168)	14% (*n* = 118)	1% (n = 5)	5% (n = 47)
Time to public health facility				
Less than 30 mins	33% (*n* = 390)	52% (*n* = 77)	43% (*n* = 242)	16% (*n* = 71)
30 mins to 1 h	38% (*n* = 449)	28% (*n* = 42)	35% (*n* = 198)	46% (*n* = 209)
1–2 h	15% (*n* = 174)	10% (*n* = 15)	14% (*n* = 82)	17% (*n* = 77)
2 h to half a day	7% (*n* = 78)	2% (*n* = 3)	2% (*n* = 12)	14% (*n* = 82)
More than half a day	1% (*n* = 8)	0% (*n* = 0)	1% (n = 3)	1% (*n* = 5)
Don’t know/ refused	6% (*n* = 68)	7% (*n* = 11)	5% (n = 30)	6% (*n* = 27)
Method of transport to health facility				
Mechanized^a^	52% (*n* = 580)	17% (*n* = 24)	54% (*n* = 289)	60% (*n* = 267)
Non-mechanized	11% (*n* = 124)	7% (*n* = 10)	0% (*n* = 1)	25% (*n* = 113)
Walking	37% (*n* = 417)	76% (*n* = 108)	46% (*n* = 243)	15% (*n* = 66)

^a^ Private car (with or without payment), public bus/ mini-van, motorcycle

**Fig. 1:**
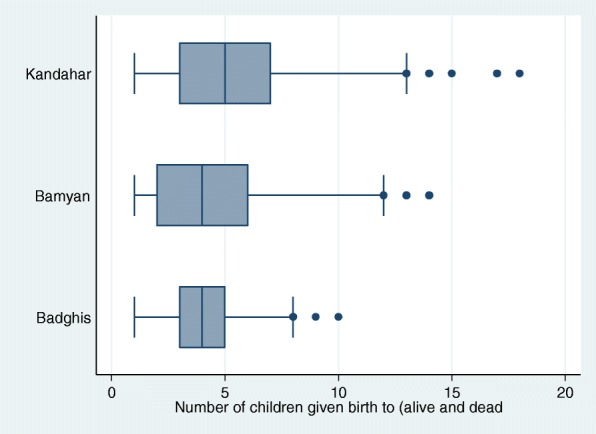
Live and still births by province (mean, minimum and maximum)

On average, women had given birth to less than five children over their lifetime (mean *n* = 4.5). Birth rates were slightly higher in Kandahar compared to the other two provinces, with a wider range including three women who had birthed 15 or more children (Fig. 1). Bagdhis province showed a comparatively smaller range of births.

An average family size of 7.9 persons was reported, which is slightly larger than national averages from the 2013–2014 Afghanistan Living Conditions Survey (7.4) though in line with national estimates of average rural household size (8.2) as reported in the 2015 Afghanistan Demographic and Health Survey [3, 6].

### Service use

Over 90% of respondents had visited a public health center at least one time in the past year. Almost half of respondents visited a public health facility between 1 and 5 times in the past year, with variation across the three provinces (Table 2). Of those who had visited a health center in the past year, the majority (60%) reported that a one-way trip was less than 30 km; however 28% responded that they did not know or provided no response, likely because they were not able to estimate distance to the nearest health facility.

Among women who had visited a public health facility in the past month, 86% (*n* = 1013) indicated that the health facility was less than two hours away (Table 2).

Over half of all women (52%) reported using mechanized transport (car or motorbike) to go to a health facility. More than one-third (37%) reported walking, and one in ten (11%) reported using a non-mechanized method (likely donkey) (Table 2). In study areas of Badghis province, there was noticeably less mechanized transport (17%), compared to more than 50% of respondents in Bamyan and Kandahar. More than three-quarters (76%) of respondents in Badghis indicated relying on walking to reach the nearest public health facility.

**Fig. 2:**
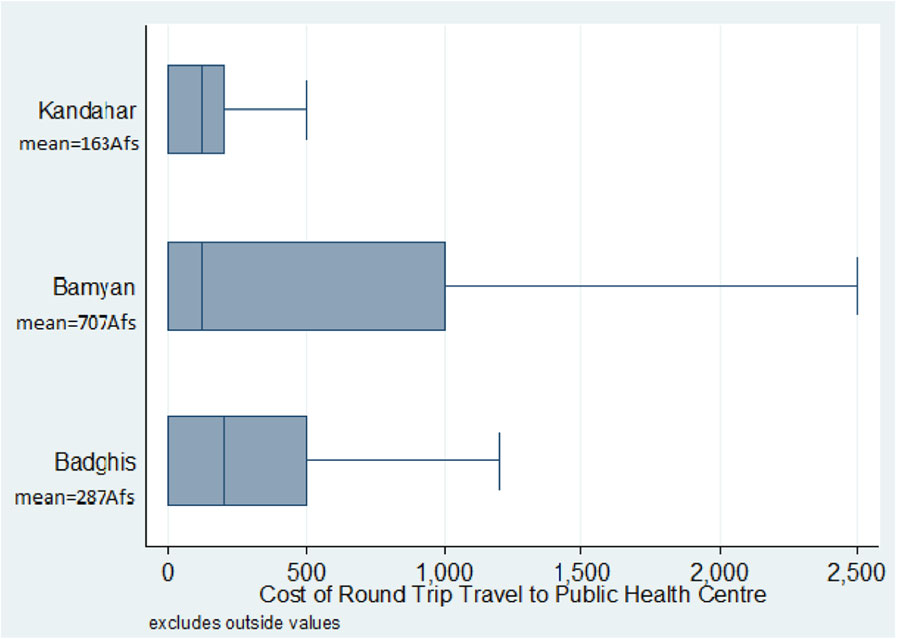
Transport costs among respondents who travelled to a health facility between 1 and 5 times per year

**Fig. 3:**
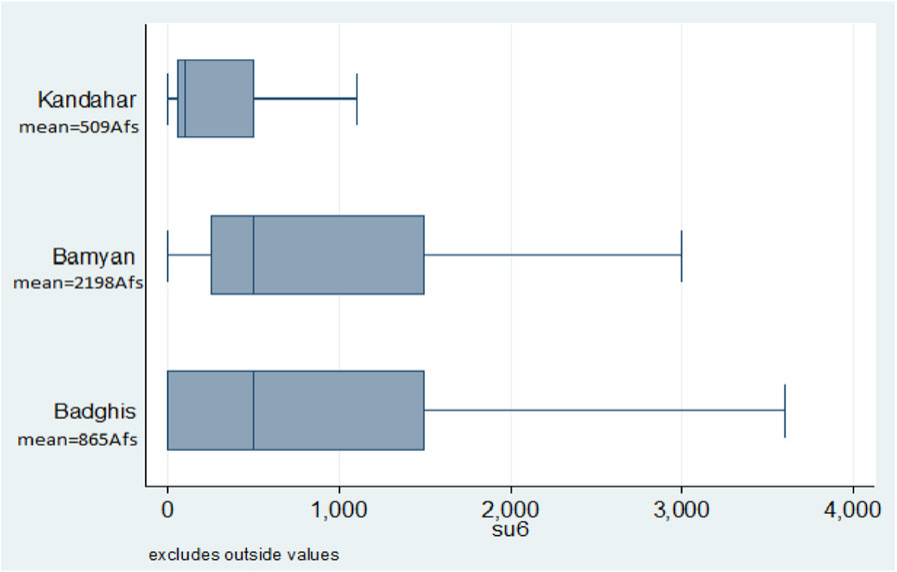
Health service costs among respondents who had travelled to a health facility between 1 and 5 times per year

On average, respondents in Kandahar and Badghis reported much lower transport and health service costs as compared to respondents in Bamyan. Interpreting the validity of cost estimates can be difficult, particularly when rural Afghan women, who are not generally in charge of payment and may lack numeracy skills, are asked for this information. This may help to explain high variation in cost estimates, ranging from 0 to 80,000 Afs. Figure 2 shows mean ranging from 163 Afs in Kandahar to a mean of 707 Afs in Bamyan. Figure 3 displays the variance in service costs reported by respondents who attended a public health centre between one and five times in the past year, with the widest variation occurring in Badghis.

### Barriers to service use

In all provinces, the cost of health services and lack of transportation availability were among the top two most cited barriers to accessing institutional delivery among household questionnaire respondents in all provinces. Despite some consistent trends, a majority of respondents who identified specific barriers to institutional delivery varied greatly between provinces.

Among those respondents who had delivered none of their children in a health center, money to pay for services was the top reason for lack of attendance overall (56%, *n* = 558) and across the study locations in all three provinces (66% in Badghis, 52% in Bamyan, 39% in Kandahar). No transportation available was the second most widely cited reason (37%, *n* = 368 overall; 47% Bamyan, 43% Badghis, 12% Kandahar), followed by family restrictions (*n* = 30%, *n* = 302). It is notable that family restrictions were comparatively higher in Badghis (46%, *n* = 241) and Kandahar (21%, *n* = 47), than in Bamyan (6%, *n* = 14) (Table 3). In addition, respondents in Badghis emphasised familial or cultural barriers as one of the top three barriers to institutional delivery, while respondents in other districts rarely mentioned this issue. Overall we found that the proportion of women reporting barriers to institutional delivery were 1.3–10.0 fold lower in Kandahar and Bamyan provinces than in Badghis province (Table 3).

**Table 3:** Barriers to institutional delivery among women who did not birth any children in a health centre among all respondents

	Province	Responses	Odds Ratio	95% CI	*P*-value
Money for health services	Badghis	62% (*n* = 508)	1.0		
	Bamyan	17% (*n* = 139)	0.110	0.092–0.145	< 0.001
	Kandahar	30% (n = 247)	0.750	0.209–0.316	< 0.001
	Overall	36% (*n* = 894)			
Money for transportation	Badghis	26% (*n* = 209)	1.0		
	Bamyan	6% (*n* = 54)	0.196	0.142–0.270	< 0.001
	Kandahar	27% (*n* = 223)	1.097	0.879–1.369	0.409
	Overall	20% (*n* = 486)			
No transportation available	Badghis	44% (*n* = 355)	1.0		
	Bamyan	16% (*n* = 137)	0.248	0.197–0.313	< 0.001
	Kandahar	13% (*n* = 107)	0.194	0.152–0.249	< 0.001
	Overall	24% (*n* = 599)			
Too far/no time	Badghis	28% (*n* = 229)	1.0		
	Bamyan	9% (*n* = 74)	0.249	0.188–0.332	< 0.001
	Kandahar	7% (*n* = 60)	0.205	0.151–0.279	< 0.001
	Overall	15% (*n* = 363)			
Not safe to travel	Badghis	13% (*n* = 102)	1.0		
	Bamyan	1% (*n* = 9)	0.075	0.037–0.150	< 0.001
	Kandahar	6% (*n* = 49)	0.446	0.312–0.637	< 0.001
	Overall	6% (*n* = 160)			
Family restrictions	Badghis	43% (*n* = 349)	1.0		
	Bamyan	2% (n = 15)	0.022	0.011–0.039	< 0.001
	Kandahar	12% (n = 102)	0.187	0.145–0.241	< 0.001
	Overall	19% (*n* = 466)			
No female staff	Badghis	13% (*n* = 103)	1.0		
	Bamyan	0% (n = 4)	0.032	0.011–0.088	< 0.001
	Kandahar	6% (*n* = 51)	0.436	0.305–0.622	< 0.001
	Overall	6% (n = 158)			
Poor quality care	Badghis	18% (*n* = 146)	1.0		
	Bamyan	2% (n = 16)	0.089	0.052–0.151	< 0.001
	Kandahar	6% (*n* = 53)	0.322	0.231–0.448	< 0.001
	Overall	9% (*n* = 215)			
Services not available/ did not know about services	Badghis	27% (*n* = 219)	1.0		
	Bamyan	4% (n = 34)	0.113	0.077–0.165	< 0.001
	Kandahar	10% (n = 81)	0.296	0.225–0.391	< 0.001
	Overall	13% (*n* = 334)			
Against religion	Badghis	3% (n = 28)	1.0		
	Bamyan	0% (n = 2)	0.066	0.015–0.278	< 0.001
	Kandahar	2% (n = 15)	0.520	0.276–0.982	0.044
	Overall	2% (*n* = 45)			
Did not think services were necessary	Badghis	20% (*n* = 159)	1.0		
	Bamyan	17% (n = 146)	0.846	0.658–1.087	0.192
	Kandahar	10% (*n* = 83)	0.424	0.316–0.569	< 0.001
	Overall	16% (*n* = 388)			

### Qualitative data

A total of 9 FGDs and 16 KIIs were conducted by trained qualitative data collectors (Table 1). Although health care services are supposed to be free in Afghanistan, payment for services was a major and recurrent theme. Qualitative data from this study confirmed and provided more detail concerning barriers to health facilities and institutional delivery, in particular relating to cost. Some respondents referred to payment for midwives and doctors as ‘sweets,’ a term often used to imply traditional pressure to give gifts for service or after happy events, such as birth, or a subtler way of asking for a bribe. A community leader from Badghis reported: “A number of people give birth to children at home because they don’t have money to pay ‘sweets’ to the midwives which is at least AFN 2,000 [about 29 USD) and this is a big amount for poor people. People who are good economically give birth to children in the clinic or hospital with midwives.”

Another common theme was distance from health facilities and problems with transport availability, unsurprising as the sample is purposively selected to represent rural areas. One woman from Kandahar reported: “The problem is transportation. Vehicles break down on the way as the road is very bad.” Problems are often confounded for rural woman, with many barriers present at once. For example, a woman from Badghis spoke of several barriers to institutional delivery: “The women face many problems during childbirth. The health centre is far and there is no ambulance to transfer the patient to the clinic, and they can’t pay for a taxi because the rent is too high.”

## Discussion

This study contributes to understanding the barriers to institutional delivery in Afghanistan in rural locations of three provinces. Overall, barriers to institutional delivery are consistent with available evidence – though with some variation by location – indicating that distance, transport cost, and social factors act as inhibitors for women to access skilled care at health facilities.

Distance to the closest health facility is an important barrier to in-facility births in rural areas in Afghanistan and in other developing countries within the region and in other regions [22, 23]. A study in Afghanistan found: “small variations in distance were still associated with major differences in skilled birth attendant use” [8]. Distance to health facilities has improved overall in the last fifteen years, with new health facilities built, staffed, and equipped, so that now 88.2% of rural populations in Afghanistan are less than two hours from a health facility [3]. Still, in areas where geographic terrain is difficult, especially in the winter months – particularly Badghis and Bamyan – maldistribution of health facilities remains an issue for access.

Associated with this finding of distance to health facilities being a barrier, transport was identified as a major obstacle for women in labour to reach a health facility. This relates to both availability of transport (mechanized and non-mechanized) and cost, particularly of mechanized transport. Potential solutions suggested by a recent systematic review include motorcycle ambulance programs, collaboration with taxi services, community education, subsidies, and vehicle maintenance [24].

Out-of-pocket spending on health in Afghanistan is high at 73% of total health expenditures [14], and especially affects rural population, who live far from facilities and generally have lower incomes. Studies in other low-income contexts demonstrated that economic status acts as a more crucial determinant than access (e.g. distance and other factors) for institutional deliveries, for example in India [25]. The wide variation in responses on costs of transport and services reported in this study make interpretation difficult as Afghan women, largely excluded from financial transactions, are less likely to be able to accurately report costs. However, women identified cost as a main barrier in the questionnaire, further supported by qualitative evidence.

One study based in Herat, the regional capital near Badghis, concluded social factors were felt to play an important role in decision-making to seek care and contributed to delays in seeking and obtaining care [18]. In this study, cultural barriers were also reported as more strongly influencing decisions around facility-based care.

This study has some limitations. First, the sample size does not account for any design effect introduced through cluster random sampling. Second, the sample within 12 districts was too small to draw nationally representative conclusions, though the sample – with almost 2500 women respondents – is larger than similar studies on maternal health conducted in Afghanistan. Variance at the community, district, and provincial levels also cannot be accurately represented with the sample size reported, though randomization did occur at village level to make the sample more representative. For each identified barrier, a chi-square test of independence was conducted to identify whether the rate of identification of this barrier was significantly different between provinces. Collecting data from female respondents, often excluded from financial transactions and with little educational exposure, likely reduces the accuracy of some data collected such as distance, time and cost of health services. Biases can occur in social desirability of responses, though this and other issues and techniques for quantitative and qualitative data collection were covered during the data collector training in efforts to minimize these biases.. The localised data collected in FGDs and KIIs is also not appropriate to be used in drawing representative conclusions about the study area as a whole. However, these data provide an additional level of insight into issues not available from quantitative information alone and are imperative to contextualizing findings and providing nuance to the evaluation of maternal and newborn service uptake, a complex and multidimensional process.

To further improve institutional deliveries in rural areas, schemes that provide conditional cash transfers which try to address multiple barriers of cost and distance have been examined in a number of settings. A systematic review of studies on conditional cash transfers aimed at improved maternal and newborn health outcomes, including studies from 8 countries, suggests that these programs have increased antenatal visits, skilled attendance at birth, delivery at a health facility, and tetanus toxoid vaccination for mothers and reduced the incidence of low birthweight [26]. Another study found that demand-side financing schemes can increase use of maternity services, though attention must be paid to supply-side components, implementation, and sustainability [27]. Findings from these studies will be tested during analysis of endline data which will be examined shortly.

## Conclusion

There are major barriers a woman to have a safe delivery in Afghanistan. These include distance, cost, and social factors. Proven and promising approaches to overcome these barriers to institutional delivery in Afghanistan should be explored and studied. While the main issues are largely the same, the nuances/local conditions need to be explored to tailor programs appropriately given Afghanistan’s varied ethnic, geographic, religious, and security makeup.

## Additional file

Additional file 1: Household questionnaire (quantitative data collection instrument). (DOCX 44 kb)

## Abbreviations

FGD: Focus group discussion
KII: Key informant interview
