# Supplementary material for: Barriers associated with care-seeking for institutional delivery among rural women in three provinces in Afghanistan
Source: BMC Pregnancy Childbirth. 2018 Jun 18;18:246. doi: 10.1186/s12884-018-1890-2 (PMC6006744; doi:10.1186/s12884-018-1890-2)
Supplement: Supplementary file 1 — Household questionnaire (quantitative data collection instrument). (DOCX 44 kb) [file 12884_2018_1890_MOESM1_ESM.docx]

# Household Questionnaire

| **INTERVIEW IDENTIFICATION** | | | | | | |
| --- | --- | --- | --- | --- | --- | --- |
|  | | | | | | |
| I1. Interviewer Code | | | \|___\|___\| | | | |
| I2. Interviewer Name | | | _____________________ | | | |
| I3. Day / Month | | | \|___ \|___ / \|___\|___ \| | | | |
| I4. Time of Interview -- Start | | | \|___ \|___\| : \|___\|__ \| AM PM | | | |
| I5. Time of Interview – End | | | \|___ \|___\| : \|___\|__ \| AM PM | | | |
| I6. Province: | | | 1. Kandahar 2. Bamyan 3. Badghis | | | |
| I7. District Name | | | - 1. Arghandab   2. Spin Boldak   3. Dand   4. Daman   5. Punjab   6. Saighan   7. Waras   8. Kahmarad   9. Qades   10. Jowand   11. Abkamary   12. Moqur | | | |
| I8. Village Name | | | ______________________ | | | |
| I9. Village Number (see code) | | | \|___\|___\|___\|___\| | | | |
| I10. Household Code | | | \|  \|  \|  \| \| --- \| --- \| --- \| | | | |
| **INTERVIEWER INTRODUCTION** | | | | | | |
| Instructions:  (1) Read Informed Consent and OBTAIN VERBAL CONSENT  **(2) Must complete all coding before you leave the household**  **Guidance notes for introducing yourself and the purpose of the interview**  Greet the interviewee according to the local culture of the area.    My name is _______________ I work for an Afghan independent research agency here in Afghanistan. We are working with the government to understand more about maternal health care for women in Afghanistan. I would like to ask one of the female members of the household some questions about what the services available to pregnant women and on which services you have used. Everything you say here today is confidential and your name or details will not be revealed to anyone.  **May I continue with the interview?**  **1) Yes**  **0) No………. End interview and replace the household** | | | | | | |
| A1. Beneficiary Present: Is there a female that lives in this household meeting the following criteria:   - Married between the ages of 15 and 49 - Has had a child that is 2 years or less - Lives in the household permanently, not visiting a household as a guest   *(If the respondent answers “No,” “Don’t Know,” or “Refuse” end the survey and replace the household)* | | | 1. Yes 2. No   98. Don’t Know  99. Refuse | | | |
| A2. I would like to speak to an eligible woman-is this OK? | | | 1. Yes 2. No | | | |
| A3. Is this a replacement household?  *(If answer is “No,” enter “97” for A4)* | | | 1. Yes 2. No | | | |
| A4. Why was the previous household replaced? | | | 1. There are no women that meet the eligibility criteria living in the household (females aged 15-49, married with a child born in the last two years) 2. There are no eligible women that meet the criteria that are home 3. The respondent does not give consent for the survey to continue 4. The respondent does not give consent for the eligible woman to answer the survey 5. The household is vacated, destroyed or neighbours say that the residents will be gone for a long period of time 6. Partly completed   97. Not asked | | | |
| **MODULE 1: RESPONDENT SELECTION** | | | | | | |
| HL1: HOW MANY PEOPLE LIVE IN THIS HOUSEHOLD? | \|___\|___\| | | | | | |
| Hl2: LISTING OF ALL ELIGIBLE RESPONDENTS   - Married between the ages of 15 and 49 - Has had a child that is 2 years or less - Lives in the household permanently, not visiting a household as a guest | # | NAME | | AGE | MARRIED | Last child born |
|  |  |  | | ___ ___ | 1. Yes 2. No | 1. Less than 1 year ago 2. Less than two years ago |
|  |  |  | | ___ ___ | 1. Yes 2. No | 1. Less than 1 year ago 2. Less than two years ago |
|  |  |  | | ___ ___ | 1. Yes 2. No | 1. Less than 1 year ago 2. Less than two years ago |
|  |  |  | | ___ ___ | 1. YES   1. NO | 1. Less than 1 year ago 2. Less than two years ago |
| HL3: If there is a respondent with a child born a year or less ago, choose that respondent  If not, go to the next respondent that has had a child in the past two years and select one randomly | NAME OF SELECTED INTERVIEWEE  _______________________________________________ | | | | | |

| **MODULE 2: WOMEN’S SURVEY** |  |
| --- | --- |
| **DEMOGRAPHICS** | |
| WB1. WHEN WERE YOU BORN?      *(Cross check with HL2)* | WERE YOU BORN   1. BEFORE SOVIET OCCUPATION (BEFORE 1978) 2. DURING SOVIET OCCUPATION (1978-1986) 3. DURING NAJIBULLAH’S PRESIDENCY (1986-1992) 4. DURRING RABBANI’S PRESIDENCY (1992-1996) 5. DURING TALIBAN CONTROL (1996-2001) 6. DURING HAMID KARZAI’S PRESIDENCY (2001-2014)   98. DON’T KNOW  99. REFUSE |
| WB2. Have you ever attended formal school that is not a madrasa  *(IF Respondent answers “NO,” “Don’t Know,” or “refuse” MARK “97” ON WB3 AND GO TO WB4)* | 1. Yes 2. No   98. DON’T KNOW (*Do not read out loud)*  99. REFUSE (*Do not read out loud)* |
| WB3. What is the highest level of school you completed? | 1. PRIMARY (1-6) 2. SECONDARY (7-9) 3. HIGH SCHOOL (10-12) 4. ADVANCED (12+)   97. NOT ASKED  98. DON’T KNOW (*Do not read out loud)*  99. REFUSE (*Do not read out loud)* |
| WB4. DO YOU KNOW HOW TO READ? | 1. YES 2. NO   98. DON’T KNOW (*Do not read out loud)*  99. REFUSE (*Do not read out loud)* |
| **SOCIO-ECONOMIC ASSESSMENT** | |
| INTERVIEWER GUIDE: (READ OUT LOUD) “I would now like to ask some questions about your household possessions. I hope this is not too sensitive but it is important for this study. Please know that there will be no financial incentive for answering these questions. All of the information you provide on this and all other parts of the survey will be kept confidential.” | |
| SE1. HOW MANY SLEEPING ROOMS DO YOU HAVE IN YOUR HOME | \|___\|___\|  98. DON’T KNOW  99. REFUSE (*Do not read out loud)* |
| SE2. Does your household own a bicycle?  *(A household includes ALL people living in the same social unit composed of people living together in the same dwelling--adults, children, non-relatives living inside the dwelling)* | 1. YES 2. NO   98. DON’T KNOW  99. REFUSE |
| SE3. Does your household own a Motorcycle? | 1. YES 2. NO 3. 98. DON’T KNOW 4. 99. REFUSE |
| SE4 DOES your household own animal-drawn- Cart? | 1. YES 2. NO   98. DON’T KNOW  99. REFUSE |
| SE5 DOES your household own car/truck? | 1. YES 2. NO   98. DON’T KNOW  99. REFUSE |
| SE6. Does your household own radio? | 1. YES 2. NO   98. DON’T KNOW  99. REFUSE |
| SE7. Does your household own Television? | 1. YES 2. NO   98. DON’T KNOW  99. REFUSE |
| SE8 Does your household own mobile phone? | 1. YES 2. NO   98. DON’T KNOW  99. REFUSE |
| SE9. Does this household own any livestock, herds, farm animals, or poultry? | 1. YES 2. NO   98. DON’T KNOW  99. REFUSE |
| SE10 How many of the following animals does this household have?    **If none, record ‘00’.**  If unknown, record ‘98’. | [SES 10A] milk cows and/or bulls? ______  [SES 10B] Horses and/or Camels?______  [SES 10c] donkeys and/or mules? _______  [SES 10D] Sheep and/or Goats? _______  [SES 10E] Chickens and/or Ducks? _______ |
| SE11. How many jeribs of land does this household own?  **If none, record ‘00’.**  If unknown, record ‘98’. | _____________jerib  ____________ beswa (1/20^th^ of a jerib) |
| SE12. Of this land used how much is used for  **If none, record ‘00’.**  If unknown, record ‘98’. | SE12_A. FARMING: _____jerib ___beswa  SE12_B. VINEYARDS/TREES_____jerib____Beswa |
| SES13. What kind of toilet facility do members of your household use?  DO NOT READ | 1. PIT LATRINE-WITH SLAB/COVERED PIT 2. PIT LATRINE-WITHOUT SLAB/OPEN 3. VENTILATED IMPROVED PIT LATRINE 4. FLUSH TO PIPED SEWER SYSTEM 5. FLUSH/POUR TO SEPTIC TANK/PIT 6. PUBLIC OR SHARED LATRINE 7. NO FACILITIES/FIELDS/GARDEN 8. OTHER (SPECIFY) ________   98. DON’T KNOW  99. Refuse |
| SE14. WHAT IS YOUR PRIMARY SOURCE OF WATER?  DO NOT READ | 1. PIPED WATER INTO DWELLING 2. HAND PUMP WITHIN HOME 3. PUBLIC TAP/STANDPIPE/HAND PIPE 4. PROTECTED DUG WELL 5. TANKER-TRUCK 6. UNPROTECTED SPRING/HAWZ 7. SURFACE WATER (STREAM/RIVER/LAKE/KAREZ) 8. OTHER (SPECIFY) ______________   98. DON’T KNOW  99. REFUSE |
| SE15. WHAT IS THE MAIN MATERIAL OF EXTERIOR WALLS  Do not read -- Record Observation | 1. NO WALLS 2. MUD WALL 3. UNCOOKED BRICKS 4. STONE 5. BAKED BRICKS 6. OTHER (SPECIFY)______________   98. DON’T KNOW |
| SE16. **Main** material of the roof.  Do not read -- Record Observation | 1. EARTH/SAND (GHORAGEL) 2. MUD PLASTER 3. CEMENT 4. CERAMIC TILES 5. TAR (ISOGAM) 6. CORRUGATED IRON (AHANPOSH) 7. OTHER (SPECIFY) ____________   98. DON’T KNOW |
| **CHILDBEARING HISTORY** | |
| CH1. How many children have you given birth to (including any who may have died soon after birth or been stillborn)? | \|___\|___\|  97. NOT ASKED  98. DON’T KNOW  99. REFUSE |
| CH2. Out of these children how many were delivered…(read each out loud)  **Total number of children delivered should match number reported in CH1**  (*If all children were delivered in a public or private health centre, mark 97 for CH3)* | 1. At home with a traditional birth attendant or family members_________________ |
|  | 1. At home with a skilled birth attendant ______________ |
|  | 1. In a public health centre ________________ |
|  | 1. In a private health centre _______________ |
|  | 1. Other ___________________ |
| CH3. IF YOU DID NOT GO TO A HEALTH CENTRE TO DELIVER YOUR LAST CHILD, WHAT WERE THE REASONS  (MAY SELECT MORE THAN ONE-DO NOT READ ANSWERS ALOUD) | 1. Did not have money for health services 2. did not have money for transportation 3. no transportation available 4. too far to travel/no time 5. not safe to travel 6. My family does not allow it 7. There are no female staff 8. quality of care is poor 9. the services are not available/i did not know about the services 10. it is against my religion 11. Did not think it was necessary 12. other (specify) _____________________   97. NOT ASKED (delivered all children in a public or private health centre)  98. Don’t know  99. refuse |
| CH4. How many children do you have? (children alive today even if they do not live with you) | \|  \|  \| \| --- \| --- \|   98. DON’T KNOW  99. REFUSE |
| **MATERNAL HEALTH SEEKING BEHAVIOUR** | |
| **ANC** | |
| anc1. In your last pregnancy (before this one – if currently pregnant) did you receive any advice before delivery, also called ante natal care?  if yes, mark according to whether she has her card present-ask to see it)  **IF NO GO Mark 97 for anc 2 and anc 3** | 1. YES HAVE CARD 2. YES, NO CARD 3. NO   97. NOT ASKED  98. DON’T KNOW  99. REFUSE |
| anc2. how many anc visits did you make during your last pregnancy at your main source of anc information?  **DO NOT READ OUT LOUD** | 1. 1 2. 2 3. 3 4. 4   5. More than 4 (Specify) ______________  97. NOT ASKED (NO ANC VISITS)  98. DON’T KNOW  99. REFUSE |
| ANC3. WHERE DID YOU RECEIVE your main source of ANC?  **DO NOT READ OUT LOUD**  **Mark only one answer** | 1. PUBLIC HEALTH CENTRE 2. PRIVATE HEALTH CENTRE 3. IN OWN OR OTHERS’ HOME-FROM SKILLED MIDWIFE OR TRADITIONAL BIRTH ATTENDANT 4. IN OWN OR OTHERS HOME FROM CHW 5. IN OWN OR OTHERS’ HOME-FROM FAMILY OR FRIENDS 6. OTHER (SPECIFY) ________________   97. NOT ASKED (NO ANC VISITS)  98. DON’T KNOW  99. REFUSE |
| anc4. DURING THIS PREGNANCY, WERE YOU GIVEN AN INJECTION IN THE ARM TO PREVENT THE BABY FROM GETTING TETANUS, THAT IS, CONVULSIONS AFTER BIRTH?  *If no skip to HSB7*  *Note: Check vaccine card* | 1. NO 2. YES – the card present 3. YES – the card not present   97. NOT ASKED  98. DON’T KNOW  99. REFUSE |
| anc5. if you did not receive any anc for your last pregnancy, what were the reasons? (may choose more than one)  Mark 97 if respondent did receive anc for last pregnancy  **do not read out loud** | 1. Did not have money for health services 2. did not have money for transportation 3. no transportation available 4. too far to travel/no time 5. not safe to travel 6. My family does not allow it 7. There are no female staff 8. quality of care is poor 9. the services are not available/i did not know about the services 10. it is against my religion 11. did not think services were necessary 12. other (specify) _____________________   97. NOT ASKED (DID RECEIVE ANC)  98. Don’t know  99. refuse |
| **POST NATAL CARE** | |
| pnc1. In your last pregnancy (before this one – if currently pregnant) did you receive advice after delivery, also called post natal care?  Note if respondent has card, ask to see it  **IF NO MARK 97 FOR PNC2 AND PNC3** | 1. NO 2. YES – the card present 3. YES – the card not present   97. NOT ASKED  98. DON’T KNOW  99. REFUSE |
| pnc2. how many PNC visits did you make after giving birth to your last child from your main source of pnc? | 1. 1 2. 2 3. 3 4. 4   5. More than 4 (SPECIFY) __________  97. NOT ASKED (NO PNC VISITS)  98. DON’T KNOW  99. REFUSE |
| pnc3. Where did you receive your main source of pnc?  **DO NOT READ OUT LOUD**  **mark only one answer** | 1. PUBLIC HEALTH CENTRE 2. PRIVATE HEALTH CENTRE 3. IN OWN OR OTHERS’ HOME-FROM SKILLED MIDWIFE OR TRADITIONAL BIRTH ATTENDANT 4. IN OWN OR OTHERS’ HOME FROM CHW 5. IN OWN OR OTHERS’ HOME-FROM FAMILY OR FRIENDS 6. OTHER (SPECIFY) ________________   97. NOT ASKED (NO PNC VISITS)  98. DON’T KNOW  99. REFUSE |
| pnc4. why did you not seek any Pnc during your last pregnancy?  (mark 97 if respondent did receive PNC)  (MAY SELECT MORE THAN ONE OPTION)  **DO NOT READ OUT LOUD** | 1. Did not have money for health services 2. did not have money for transportation 3. no transportation available 4. too far to travel/no time 5. not safe to travel 6. My family does not allow it 7. There are no female staff 8. quality of care is poor 9. the services are not available/i did not know about the services 10. it is against my religion 11. Did not feel services were necessary 12. other (specify) _____________________   97. NOT ASKED (RECEIVED PNC)  98. Don’t know  99. REFUSE |
| **COMMUNITY HEALTH WORKER AND INCENTIVES** | |
| CHW1. HAVE YOU EVER MET WITH OR BEEN VISITED BY A COMMUNITY HEALTH WORKER to discuss health issues? (this excludes social or other visits not relating to health) | 1. YES 2. NO   98. DON’T KNOW  99. REFUSE |
| chw2. DID YOU RECEIVE ANY COUNSELING during your pregnancy about maternal health, such as maternal nutrition, breastfeeding practices, or danger signs?  ask to see card if present | 1. YES, CARD PRESENT 2. YES, NO CARD 3. NO   98. DON’T KNOW  99. REFUSE |
| CHW3. WHO DID YOU RECEIVE THIS ADVICE FROM?  (MAY SELECT MORE THAN ONE OPTION)  IF did not SELECT COMMUNITY HEALTH WORKEr mark ‘97’ for chw4 AND CHW5 | 1. COMMUNITY HEALTH WORKER 2. HEALTH CENTRE STAFF 3. FAMILY 4. TRADITIONAL BIRTH ATTENDANT 5. OTHER (SPECIFY) ________________________   98. DON’T KNOW  99. REFUSE |
| CHW4. WHAT WAS THE sex OF YOUR CHW? | 1. FEMALE 2. MALE 3. RECEIVED ADVICE FROM BOTH MALE AND FEMALE CHW’S 4. NOT ASKED 5. DON’T KNOW 6. REFUSE |
| CHW5. WHAT TYPE OF ADVICE ABOUT YOUR PREGNANCY AND STAYING HEALTHY DID YOU RECEIVE?  (DO NOT READ THE OPTIONS) | 1. nutrition 2. danger signs of a problem 3. delivering at a facility 4. post natal care for mother and baby 5. post natal family planning 6. PRENATAL CARE AT A FACILITY 7. OTHER (SPECIFY)____________________ 8. NOT ASKED 9. DON’T KNOW 10. REFUSE |

| **SERVICE UTILISATION** | |
| --- | --- |
| su1. In the past 12 months, how often did you AND YOUR FAMILY USE A public health centre Facility? (for any reason-does not have to be for maternal health care) | 1. NONE (0) 2. 1-5 TIMES 3. 6-10 TIMES 4. 10+ TIMES 5. DON’T KNOW 6. REFUSE |
| SU2. What was the main form of transportation for your visits?  can say what they think would be available even if they have not been in the past month  IF RESPONDENT ANSWERS MORE THAN ONE, ASK WHAT THEY USE MOST FREQUENTLY  (DO NOT READ OUT LOUD) | 1. FOOT 2. OXCART OR ANIMAL (WITHOUT PAYMENT) 3. OXCART OR ANIMAL (WITH PAYMENT) 4. PRIVATE CAR (WITHOUT PAYMENT) 5. PRIVATE CAR (WITH PAYMENT) 6. PUBLIC BUS/MINI-VAN 7. BICYCLE 8. /MOTORCYCLE 9. OTHER (SPECIFY)_____________________________   98. DON’T KNOW  99. REFUSE |
| SU3. What distance do you travel to reach the public health centre facility?  can estimate even if they have not been in the past month | 1. 1 Km to 30 Km (Record number of KM) ______ 2. Greater than 30 3. DON’T KNOW   99. REFUSE |
| SU4. How long did it take you to travel to this public health facility?  can estimate even if they have not been in the past month  (do not read answer) | 1. Less than 30 minutes 2. 30 to 1 hour 3. 1 to 2 hours 4. More than 2 hours to less than ½ day 5. More than ½ day   98. DON’T KNOW  99. REFUSE |
|  |  |
| SU5. What was the cost of travel to and from this public health centre?  can say what they think it would be even if they have not been in the past month  (do not read answer)  Write the exact amount in the line provided. | Record amount in Afs   1. _________________________________AFS 2. DON’T KNOW |
| SU6. what would you estimate to be the cost of service?  Prompt: Did you pay a Registration fee? For Lab work? for medicine?  can say what they think it would be even if they have not been in the past month | Record amount in Afs  _________________________________AFS  98. DON’T KNOW  99. REFUSE |
| SU7. what would you estimate to be the value of any non-monetary gifts for service?  Enter 0 if no non-monetary costs | Record amount in Afs  _________________________________AFS   1. DON’T KNOW 2. REFUSE |
| ENUMERATOR INSTRUCTIONS: Thank you for your participation. It was very helpful and important in plans for improving maternal health in Afghanistan. I would like to ask for a contact number if that is possible. it can be family member or other relative if that is better. i would like to say again that all of your information you shared with me today will be kept confidential. thank you again! | |
| Can you please provide a contact number for me to reach you?  *If you can provide more than one that would be very good* | 1. Personal Number   _________________________________   1. Family Number   _________________________________   1. Relatives Number   _________________________________ |

**Comments:**
